# Endovascular Treatment of Medial Tentorial Dural Arteriovenous Fistula Through the Dural Branch of the Pial Artery

**DOI:** 10.3389/fneur.2021.736919

**Published:** 2021-12-13

**Authors:** Chan-Lin Chu, Yu-Cheng Chu, Chee-Tat Lam, Tsong-Hai Lee, Shih-Chao Chien, Chih-Hua Yeh, Yi-Ming Wu, Ho-Fai Wong

**Affiliations:** ^1^Department of Neurology, New Taipei Municipal Tucheng Hospital, Chang Gung Memorial Hospital, New Taipei City, Taiwan; ^2^College of Medicine, Chang Gung University, Taoyuan, Taiwan; ^3^Department of Critical Care, Far-Eastern Hospital, Taipei, Taiwan; ^4^Department of Neurosurgery, Shin Kong Wu Ho-Su Memorial Hospital, Taipei, Taiwan; ^5^Department of Neurology, Chang Gung Memorial Hospital, Taoyuan, Taiwan; ^6^Department of Emergency Medicine, Mackay Memorial Hospital, Taipei, Taiwan; ^7^Division of Neuroradiology, Department of Medical Imaging and Intervention, Chang Gung Memorial Hospital, Taoyuan, Taiwan

**Keywords:** arteriovenous fistula, tentorial, pial anastomosis, Onyx, transarterial embolization

## Abstract

**Background:** Tentorial dural arteriovenous fistula is a rare subtype of intracranial dural arteriovenous fistula (DAVF) with a deteriorating natural course, which may be attributed to its pial angioarchitecture. TDAVF often harbors feeders arising from pial arteries (FPAs). Reports have revealed that, if these feeders are not obliterated early, the restricted venous outflow during the embolization process may cause upstream congestion in the fragile pial network, which increases the risk of hemorrhagic complications. Because most reported cases of TDAVF were embolized through feeders from non-pial arteries (FNPAs), little is known of the feasibility of direct embolization through FPAs.

**Methods:** We present three patients with medial TDAVFs that were embolized through the dural branches of the posterior cerebral and superior cerebellar arteries. Findings from brain magnetic resonance imaging, computed tomography, angiography, and clinical outcomes are described. Furthermore, we performed a review of the literature on TDAVFs with FPAs.

**Results:** The fistulas were completely obliterated in two patients; both recovered well with no procedure-related complications. The fistula was nearly obliterated in one patient, who developed left superior cerebellum and midbrain infarct due to the reflux of the embolizer into the left superior cerebellar artery. Including our cases, eight cases of TDAVFs with direct embolization through the FPAs have been reported, and ischemic complications occurred in three (37.5%).

**Conclusions:** Advancing microcatheter tips as close to the fistula point as possible and remaining highly aware of potential embolizer flow back into the pial artery are key factors in achieving successful embolization. Balloon-assisted embolization may be an option for treating TDAVFs with FPAs in the future.

## Introduction

Intracranial dural arteriovenous fistulas (DAVFs) account for 10–15% of intracranial vascular shunts (1). Among intracranial DAVFs, tentorial DAVF (TDAVF) is a rare subtype, which accounts for 4–8% of intracranial DAVFs (2). Compared with other intracranial DAVFs, the natural history of TDAVFs is generally marked by deterioration (3), which mandates early interventional treatment. Treatment choices for intracranial DAVFs include surgical devascularization, radiotherapy, and endovascular treatment. Because of the deep location and complex angioarchitecture of TDAVFs, they are less amenable to surgical treatment; therefore, transarterial embolization (TAE) is gaining popularity as a first-line treatment for TDAVFs (1, 4–7). Feeders from pial arteries (FPAs) are common in TDAVFs (8), and their presence has been associated with periprocedural ischemic and hemorrhagic complications (7, 9); possible reasons for this include penetration of embolizers into the segment of the pial artery, supplying blood to the brain parenchyma, which causes cerebral ischemia, and restriction of the venous outlet during embolization, which causes congestion in the upstream pial network and subsequently, hemorrhage.

In most reports, treated TDAVFs were embolized through feeders from non-pial arteries (FNPAs). In this report, we present our experience of direct embolization through the dural branches of pial arteries in three patients with medial TDAVF and review the literature on views on and treatment of TDAVFs with FPAs.

## Materials and Methods

This retrospective study was conducted in accordance with the Declaration of Helsinki; it was approved by the Institutional Review Board of Chang Gung Memorial Hospital (IRB: 202100129B0). Three patients with TDAVFs underwent TAE in 2020 after providing informed consent.

### Description of Technique

The procedure was conducted through bifemoral arterial access under general endotracheal anesthesia with heparinization. A 5F guide catheter (Envoy, Codman, Raynham, MA, USA) or a triaxial guide catheter system consisting of a 6F guide catheter (Neuron 088, Penumbra, Alameda, CA) and 5F intermediate catheters (Navien, Covidien, Paris, France; Sofia, Microvention, Stanford, CA, USA) was positioned at the vertebral artery. An angiographic catheter was positioned at the carotid artery or the contralateral vertebral artery for a control angiogram. Under road-mapping guidance, the FPAs were super selected with a microcatheter (Apollo, Medtronic, Singapore, Singapore; Marathon, Medtronic, Singapore, Singapore; Echelon-10, Medtronic, Singapore, Singapore) over a 10-inch micro guide wire (Transend, Boston Scientific, Botany, Australia) as close to the fistula point as possible. After the micro guide wire was removed, a contrast medium was injected through the microcatheter to confirm the proximity of the microcatheter tip to the fistula and that the brain parenchyma was not stained. Embolization was performed by injecting ethylene vinyl alcohol copolymer (Onyx-18, Medtronic, Singapore, Singapore) through the microcatheter using the “plug and push” technique until the fistula was obliterated or flowback or penetration of Onyx to undesired vessels occurred, which was confirmed with regular angiography performed through the intermediate catheter, guiding catheter, or angiographic catheter.

### Illustrative Cases

#### Case 1

A 57-year-old man with hypertension presented with intermittent weakness of the left limbs (with muscular strength as low as Grade 4 on the Medical Research Council scale), a left facial burning sensation, and left upper limb numbness persisting for 3 weeks. Brain magnetic resonance angiography (MRA) revealed serpentine vessels at the quadrigeminal cistern. Angiography revealed a DAVF at the edge of the tentorial incisura that was draining mainly through the vein of Galen to the straight sinus and partly draining through the cerebellar cortical vein to the transverse sinus. The feeders were the bilateral occipital arteries, right middle meningeal artery (MMA), marginal tentorial artery from the right internal carotid artery (ICA), dural branch of the bilateral posterior cerebral arteries (PCAs), and left artery of the falx cerebelli.

**Figure 1:**
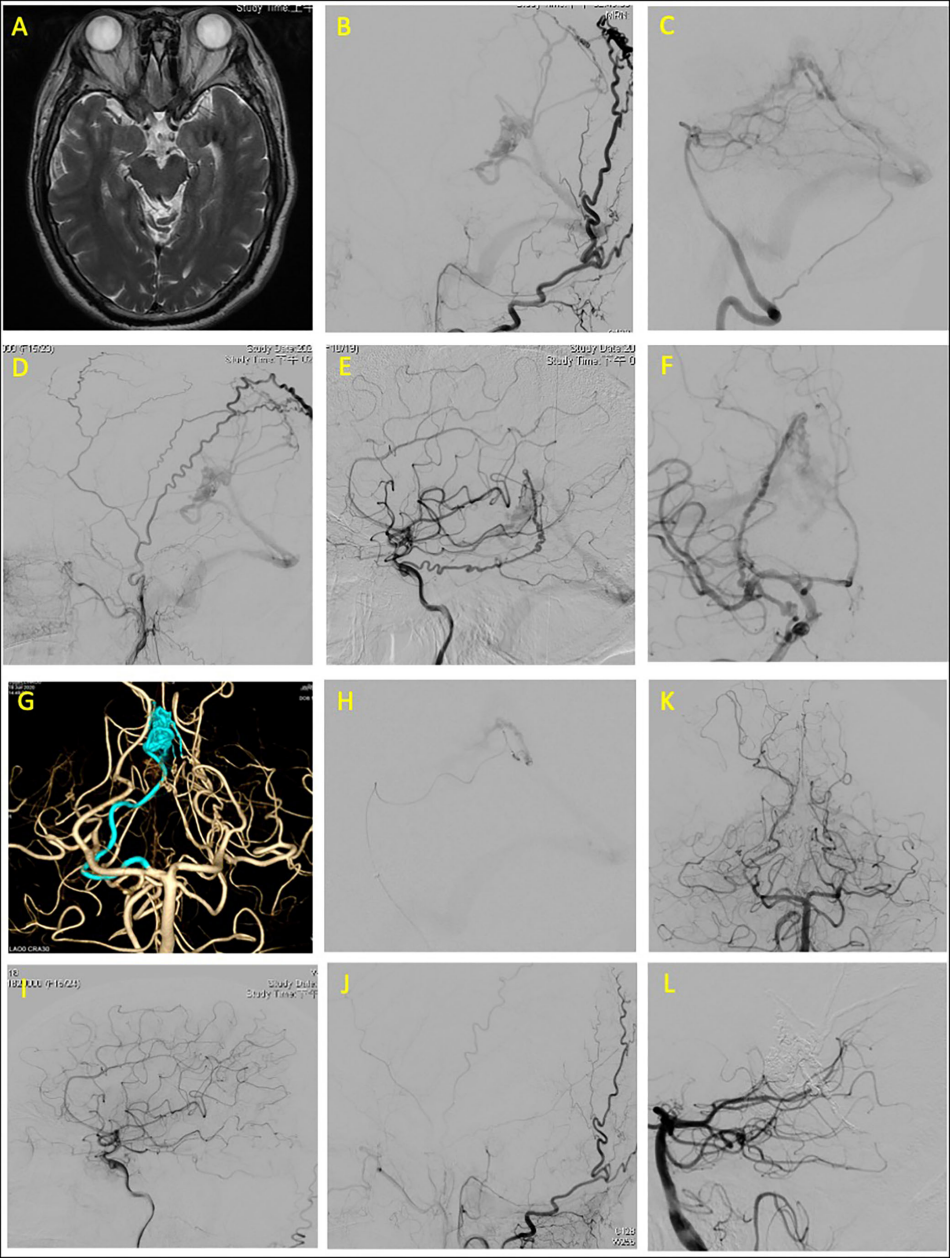
Tentorial dural arteriovenous fistula of case 1. **(A)** Brain magnetic resonance angiography (MRA) revealed serpentine vessels at the quadrigeminal cistern. Angiograms of **(B)** left external carotid artery (ECA), **(C)** left vertebral artery (VA), **(D)** right external carotid artery, and **(E,F)** right internal carotid artery (ICA) revealed that the feeding arteries were the left middle meningeal artery, bilateral occipital arteries, right marginal tentorial artery, bilateral artery of Davidoff and Schechter (ADS), and left artery of falx cerebelli. **(G)** Three-dimensional (3D) reconstruction of right ADS, which was superselected **(H)** for embolization. Total obliteration of fistula confirmed through postprocedural angiogram from **(I)** right ICA, **(J)** left ECA, and **(K,L)** left VA.

The dural branch of the P1 segment of the right PCAs was superselected with a Marathon microcatheter to approach the fistula point at the falx cerebelli. Embolization was performed with 3-ml Onyx to complete obliteration of the fistula. Post-embolization angiography images revealed complete obliteration of the patient's fistula (Figure 1).

The subsequent course of the patient was uneventful after the operation; the muscle strength of the left side improved to Grade 5 on the Medical Research Council scale. The patient had a modified Rankin Scale (mRS) score of 1 at 9-month follow-up.

#### Case 2

A 49-year-old woman with history of diabetes mellitus reported sudden-onset severe headache and dizziness. Brain computed tomography revealed diffuse subarachnoid hemorrhage. Angiography revealed a TDAVF with feeders from the bilateral superior cerebellar arteries (SCAs), left posterior meningeal artery, and bilateral occipital arteries. The TDAVF was draining through a varix in the draining vein at the tentorial edge of the vein of Galen.

**Figure 2:**
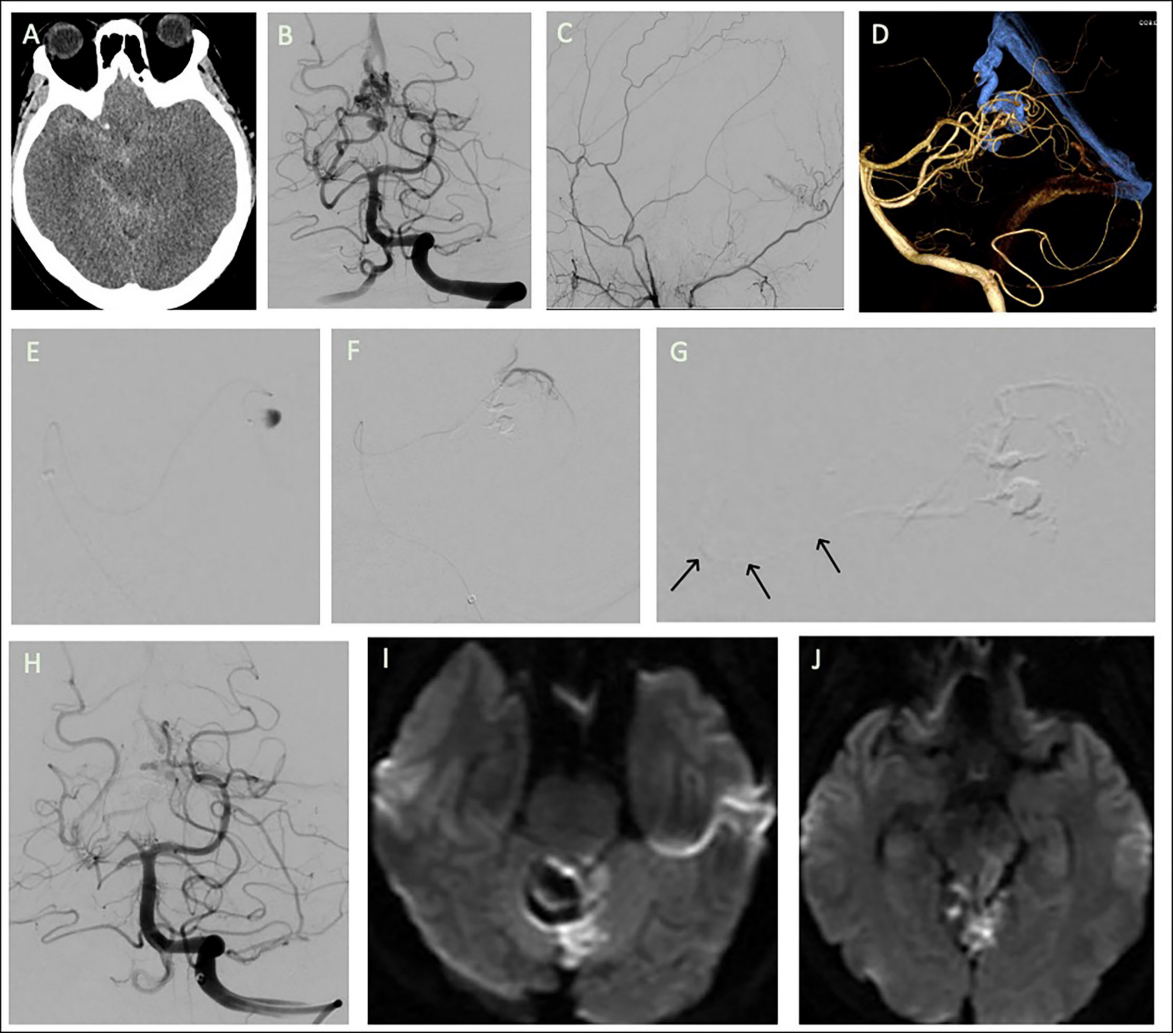
Tentorial dural arteriovenous fistula of case 2. **(A)** Brain computed tomography revealed diffuse subarachnoid hemorrhage. Angiograms of **(B)** left vertebral artery (VA) and **(C)** right external carotid artery. The feeders were the bilateral superior cerebellar arteries (SCAs), bilateral occipital arteries, and left posterior meningeal artery. **(D)** 3D reconstruction of the fistula. Embolization was performed after navigation of a microcatheter from right SCA to the **(E)** varix in the draining vein, with most of the fistula being obliterated. The microcatheter then approached the fistula from **(F)** the left SCA for further embolization; **(G)** inadvertent flowback of the embolizer to the SCA orifice was noted (arrows). **(H)** Post-embolization angiogram of left VA injection. **(I,J)** Follow-up brain MRA revealed acute infarction at tegmentum and tectum of left midbrain and left superior cerebellum.

The right medial tentorial artery from the right SCA was superselected with an Apollo microcatheter close to the fistula at the varix. Embolization was performed with Onyx to obliterate most of the fistula. A Marathon microcatheter was then navigated from the left SCA to the left medial tentorial artery to perform further embolization. However, during the second embolization, a reflux of Onyx to the left SCA orifice was noted (Figure 2).

The patient had right hemiplegia after intervention. Emergent brain MRA revealed acute infarction in the left superior cerebellum and the tectum of the midbrain. At 6-month follow-up, the patient still presented with right hemiplegia and had an mRS score of 4.

#### Case 3

**Figure 3:**
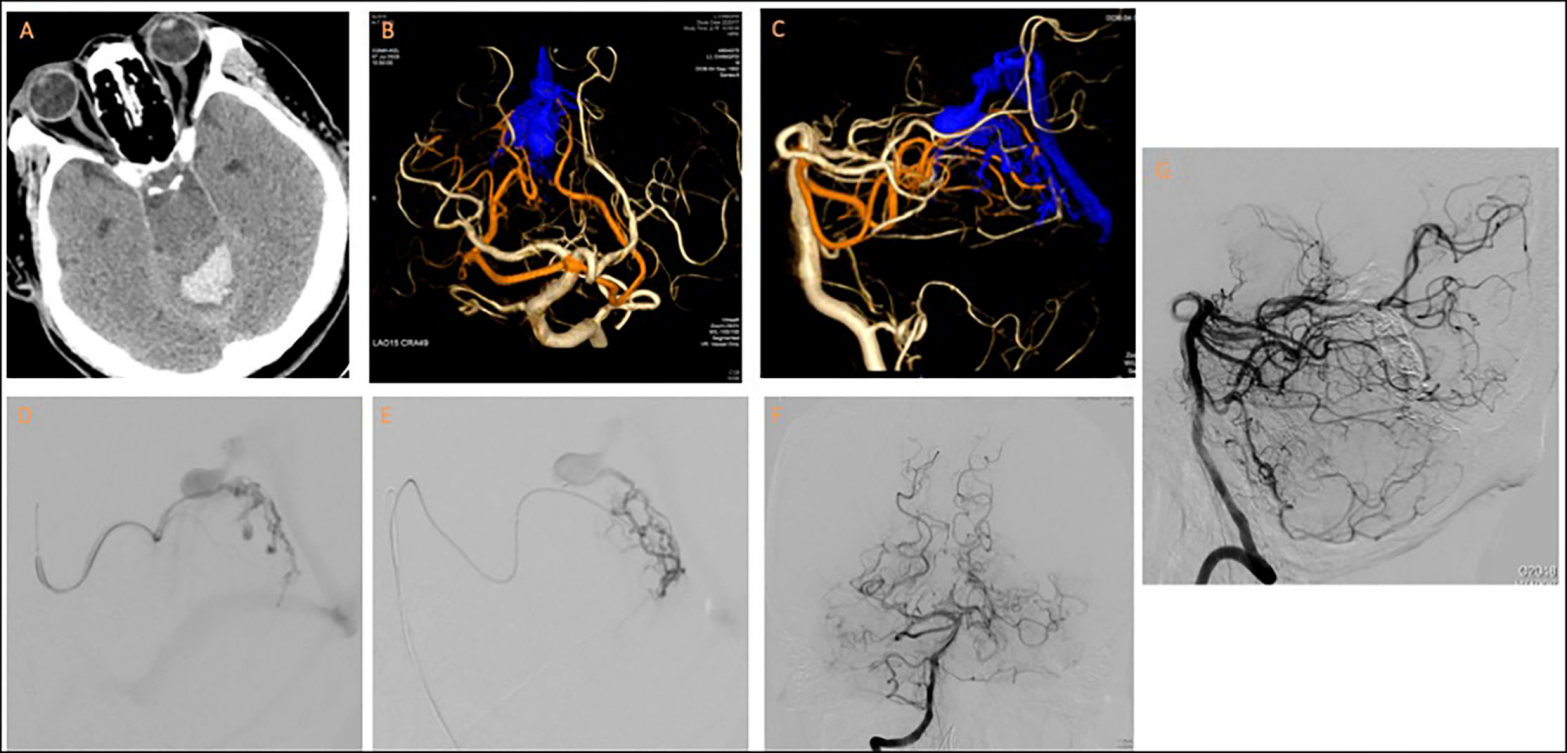
Tentorial dural arteriovenous fistula of case 3. **(A)** Brain computed tomography revealed fourth-ventricle intraventricular hemorrhage. **(B,C)** 3D reconstruction of cerebral angiogram revealed the fistula was supplied by medial tentorial branches of bilateral SCAs and drained into vein of Galen through a varix. Notably, a stricture was present at the venosinus junction. Embolization was performed from **(D)** left and **(E)** right dural branches of SCAs. Post-embolization angiogram from **(F,G)** right vertebral artery revealed total obliteration of the fistula.

A 67-year-old man with hypertension presented with sudden-onset nausea, vomiting, dizziness, dysarthria, ataxia, and general weakness. Brain computed tomography revealed intracerebral hemorrhage at the cerebellar vermis with rupture into the fourth ventricle. An external ventricular drain was placed. Angiography revealed a DAVF located at the falx cerebelli with the medial tentorial branches of the bilateral SCAs as feeders and that drained through a varix to the vein of Galen and the straight sinus. A venous stenosis was also noted at the venosinus junction. The dural branch of the left SCA was superselected with an Apollo microcatheter to approach the fistula point at the falx cerebelli; the right SCA was superselected with an Echelon-10 microcatheter. Complete obliteration of the fistula was achieved through Onyx injection (Figure 3).

The patient presented with no new neurological deficits after TAE. The patient actively participated in a post-acute care rehabilitation program and had an mRS score of 4 at 9-month follow-up.

## Discussion

In this report, we demonstrated that obliteration of TDAVF could be achieved with embolization through the dural branches of the pial arteries, the PCA [or the artery of Davidoff and Schechter (ADS)], and the SCA (or the medial tentorial branch of the SCA).

The goal of DAVF treatment is to occlude the shunting points of the fistula while minimizing the effect on the normal vascular system. A careful examination of the angioarchitecture of the TDAVF (including fistula location, characteristics of feeding arteries, draining veins and sinuses, the presence of pial artery supply and aneurysm, and the pressure and velocity of the arteriovenous shunt) should be performed before endovascular treatment is initiated to tailor treatment strategies to the patient.

A TDAVF may receive a blood supply from different locations: anteriorly from the tentorial artery; inferiorly from the PCA or SCA; superiorly from the falcine and MMA; or posteriorly from the occipital, posterior meningeal, or falx arteries (10). DAVFs with FPAs account for ~11.3% of all DAVFs (7, 10), and FPAs are even more common in TDAVFs (57%) (8).

Lawton and Tan (10) divided medial TDAVFs into Galenic, straight sinus, and torcula subtypes. In their case series, the presence of a PCA or SCA blood supply to a TDAVF was frequently pathognomonic of the Galenic subtype (present in 86% of cases), and these two feeders were found only in medial TDAVF. A study in 2018 revealed that these two arteries could also supply blood to TDAVF located at the superior petrosal and transverse-sigmoid sinuses (8). The cases of TDAVFs with FPAs treated through TAE of this study are summarized in Supplementary Table 1.

The PCA and SCA have rarely been selected as the primary access points for TAE of TDAVF because of the risk of causing brainstem and cerebellar infarction; the proximal SCA has perforating branches that supply blood to the rostral cerebellum and brainstem, and anastomotic channels exist between the collicular branch of the PCA and the medial terminal stem of the SCA (11). Therefore, to lower the risk of complications, a microcatheter should be advanced as close as possible to the fistula points to allow for a longer reflux course and to prevent penetration of the embolizer into the perforating branches. Furthermore, during embolization, clinicians should remain highly vigilant to prevent ischemic complications due to reflux of the embolizer into brain-supplying arterial segments. In our second case, this type of complication occurred; however, it should be avoidable.

Bhatia et al. (12) proposed risk-reduction strategies for the treatment of TDAVFs with an ADS blood supply; these included initial embolization from non-ADS arterial feeders to reduce competitive flow and the use of the pressure cooker technique when direct embolization is performed through the ADS to minimize reflux across the ADS. In the pressure cooker technique, two microcatheters are used; the proximal microcatheter creates a plug made of coil and cyanoacrylate, which enables the injection of non-adhesive liquid embolizer through the distal microcatheter. However, to our knowledge, this technique has only been employed in one case of TDAVF with FPA. The technique's infrequent use is likely due to the small calibers of pial arteries (~1.7–2 mm at the P2 segment of the PCA) (13), which prevent them from easily accommodating two microcatheters at once. A more practical alternative may be balloon-assisted embolization, which can be achieved with a Scepter Mini (Microvention) microballoon catheter. This extra small–sized (a distal tip outer diameter of 1.6 F), dual lumen, compliant microcatheter can be positioned more distally into the feeder. In addition to embolization, the microballoon catheter can be used in super selective flow arrest and as navigation support to facilitate treatment of intracranial arteriovenous shunts with small feeders (14). Reported cases of patients with TDAVFs embolized directly through FPAs are summarized in Table 1.

**Table 1:** Reported TDAVF patients embolized directly through the pial artery.

**Author**	**Patient age**	**Sex**	**Access**	**Fistula type**	**Presentation**	**Feeders**	**Drainage**	**Cognard type**	**Complications**
This study	57	M	PCA (R)	Galenic	Left limbs numbness and weakness, left facial burning sensation	SCA (B)	Vein of Galen	IIa+b	None
	49	F	SCA (B)	Galenic	SAH	SCA (B), posterior meningeal artery (L), OA (B)	Vein of Galen, varix	IIa+b	SCA territory infarction
	67	M	SCA (B)	Galenic	IVH	MMA (R), marginal tentorial artery (R), bilateral ADSs, falx cerebelli artery (L)	Vein of Galen, varix, cerebellar cortical veins	IIa+b	None
Huang et al. (6)	42	M	PCA (L)	Galenic	SAH/IVH	PCA, OA, MHT, MMA	Vein of Galen, varix	III	None
	65	M	SCA (L)	Galenic	SAH	SCA (L), MHT (R), OA (L), APA	Perimesencephalic Vein of Galen	III	Microcatheter retention
Wu et al. (7)	48	M	PCA (L)	Galenic	IVH	PCA, MMA, OA, PMA, MHT	Vein of Galen, Basal vein of Rosenthal	IV	None
Zhang et al. (11)	60	M	SCA (L)	Tentorium cerebelli	ICH	Bilateral MMA, SCA (L)	Basal vein of Rosenthal, dilated	IV	SCA territory infarction
	41	M	PCA (L)	Tentorium cerebelli	ICH	PCA (L)	Basal vein of Rosenthal, varix	IV	SCA territory infarction

*ADS, artert of Davidoff and Schechter; APA, ascending pharyngeal artery; ICH, intracerebral hemorrhage; IVH, intraventricular hemorrhage; MHT, meningohypophyseal trunk; MMA, middle meningeal artery; OA, occipital artery; PCA, posterior cerebral artery; PMA, posterior meningeal artery; SAH, subarachnoid hemorrhage; SCA, superior cerebellar artery*.

Wu et al. (7) suggested that the presence of a pial arterial supply in a TDAVF is a predictor of intracerebral hemorrhage complications (ICHs), which occurred in 33% (2/6) of their included patients, even when complete fistula obliteration was achieved. The proposed mechanism underlying this phenomenon is that restricted venous outflow during embolization can lead to venous hypertension and congestion of the upstream fragile pial network, which then causes ICHs. Wu et al. (7) proposed that obliteration of the FPA before fistula embolization may reduce the risk of hemorrhage. By contrast, in a case series by Osada and Krings (8) no ICHs occurred after endovascular treatment of 17 DAVFs with FPAs (eight patients with TDAVFs); however, the authors of this study did not detail the treatment strategies they employed. In addition, Hetts et al. reported that patients with intracranial DAVFs with a pial arterial supply had a higher frequency (10.3%) of ischemic stroke after endovascular treatment compared with those without a pial arterial supply (1.1%). Possible causes for this include retrograde thrombosis of the pial artery, reflux of the embolizer into the pial artery segments that supply brain parenchyma, or periprocedural hypercoagulability (9).

Osada and Krings (8) categorized DAVFs with pial arterial supplies into two types: a dilated preexisting dural branch of the pial artery (Type 1) and purely pial artery (Type 2). Type 1 has a linear course and runs along the dura, whereas Type 2 is ramified, fragile, and runs a tortuous course. According to this classification system, all of our reported FPAs are Type 1. A pathophysiological hypothesis for the formation of DAVFs with FPAs is that a high-flow shunt causes a steal phenomenon and local ischemia, which stimulates vascular endothelial growth factors and angiogenesis from the pial artery to the fistula (7).

Previous reports have generally selected the MMA, when available, for TAE of TDAVFs. The MMA runs a long course in the dura and can tolerate a long reflux of embolizer; however, the reflux should not approach the foramen spinosum, where damage to the trigeminal and facial nerves can occur if the petrosal branch of the MMA is embolized. The occipital artery is occasionally used for TAE. However, this artery is surrounded by loose connective tissue and may run a tortuous course, especially when accommodating a high flow, which renders distal catheterization difficult (4). The marginal tentorial artery, which arises from an inferolateral trunk or meningohypophyseal trunk, is occasionally used for TAE if no adequate extracranial arterial access is available; however, an inadvertent reflux of the embolizer into the ICA may cause cerebral infarct. Furthermore, damage to the oculomotor, trochlear, trigeminal, and abducens nerves could occur if the branches of the meningohypophyseal trunk are embolized (15).

Transvenous embolization may be considered if the microcatheter can be navigated to the fistula point. However, we did not attempt transvenous embolization because the presence of stricture or a tortuous course hampered easy access to the fistula points. Moreover, partial occlusion in the draining vein may precipitate venous hypertension and create a risk of hemorrhage in the upstream pial network. Therefore, transvenous embolization may serve as an alternative treatment or a combined treatment with TAE for TDAVFs (11, 16).

## Conclusions

Tentorial dural arteriovenous fistulas are deeply located DAVFs with a deteriorating natural course; they are frequently supplied blood by branches of the PCA or SCA, which can be directly catheterized for TAE to achieve complete obliteration of fistulas. Advancing microcatheter tips as close to the fistula point as possible and remaining highly aware of potential embolizer reflux are key factors in achieving successful embolization. Furthermore, balloon-assisted embolization through FPAs may be an option for treating TDAVFs in the future.

## Data Availability Statement

The raw data supporting the conclusions of this article will be made available by the authors, without undue reservation.

## Ethics Statement

The studies involving human participants were reviewed and approved by Institutional Review Board at Chang Gung Memorial Hospital. Written informed consent for participation was not required for this study in accordance with the national legislation and the institutional requirements. Written informed consent was obtained from the individual(s) for the publication of any potentially identifiable images or data included in this article.

## Author Contributions

C-LC and Y-CC: conceptualization, investigation, writing—original draft, review, and editing. C-TL, S-CC, C-HY, and Y-MW: writing—review and editing. T-HL: writing—review and editing and supervision. H-FW: conceptualization, resources, writing—review and editing, and supervision.

## Conflict of Interest

The authors declare that the research was conducted in the absence of any commercial or financial relationships that could be construed as a potential conflict of interest.

## Publisher's Note

All claims expressed in this article are solely those of the authors and do not necessarily represent those of their affiliated organizations, or those of the publisher, the editors and the reviewers. Any product that may be evaluated in this article, or claim that may be made by its manufacturer, is not guaranteed or endorsed by the publisher.

## Abbreviations

ADS: artery of Davidoff and Schechter
APA: ascending pharyngeal artery
CT: computed tomography
DAVF: dural arteriovenous fistula
ECA: external carotid artery
FNPA: feeders arising from non-pial artery
FPA: feeders arising from pial artery
ICA: internal carotid artery
ICH: intracerebral hemorrhage
IVH: intraventricular hemorrhage
MHT: meningohypophyseal trunk
MMA: middle meningeal artery
MRA: magnetic resonance angiography
OA: occipital artery
PCA: posterior cerebral artery
PMA: posterior meningeal artery
SAH: subarachnoid hemorrhage
SCA: superior cerebellar artery
TAE: transarterial embolization
TDAVF: tentorial dural arteriovenous fistula
VA: vertebral artery.
