# Supplementary material for: Endovascular Treatment of Medial Tentorial Dural Arteriovenous Fistula Through the Dural Branch of the Pial Artery
Source: Front Neurol. 2021 Dec 13;12:736919. doi: 10.3389/fneur.2021.736919 (PMC8710483; doi:10.3389/fneur.2021.736919)
Supplement: Supplementary file 1 [file Table_1.DOCX]

| **Supplementary Table S1** Reported cases with tentorial dural arteriovenous fistulas with feeders arising from pial arteries treated by transarterial embolization | | | | | | | | | | |
| --- | --- | --- | --- | --- | --- | --- | --- | --- | --- | --- |
| Author | Age | Sex | Location | Presentation | Feeders | Drainage | Cognard type | Complication | Outcome | Type of pial artery supply |
| Chu et al. | 57 | M | Galenic | Left limbs numbness and weakness, left facial burning sensation | OA (B), MMA (R), marginal tentorial branch of ICA (R), falx cerebelli artery (L) | VOG, cerebellar leptomeningeal veins | IIa+b | None | Complete obliteration | Dilated dural branch (PCA) |
|  | 49 | F | Galenic | SAH | SCA (B), posterior meningeal artery (L), OA (B) | Vein of Galen, varix | Iia+b |  |  | Dilated dural branch (SCA) |
|  | 67 | M | Galenic | IVH | SCA (B) | VOG (varix) | Iia+b |  |  | Dilated dural branch (SCA) |
| Kortman et al.^1^ | 50 | M | Tentorium cerebelli | Occipital hemorrhage | SCA medial branch |  | III | Post procedural pain in 83% of total cases | All were completely occluded |  |
|  | 63 | M | Tentorium cerebelli | Cerebellar hemorrhae | PICA, MHT, PMA, and OA |  | IV |  |  |  |
|  | 68 | M | Tentorium cerebelli | Cerebellar hemorrhae | PMA, SCA, OA, and MHT |  | IV |  |  |  |
|  | 56 | M | Tentorium cerebelli | Cerebellar hemorrhae | PM, SCA, MHT and MMA |  | IV |  |  |  |
|  | 44 | M | Tentorium cerebelli | Cerebellar hemorrhae | PMA, SCA, OA and MHT |  | IV |  |  |  |
| Liu et al.^2^ | 55 | M | Medial | Vertigo, convulsion | MMA, OA, SPA, PMA | Cerebellar leptomeningeal venous aneurysm | Borden III | None | Among the 6 patients reported, 66.7% were totally obliterated; 33.3% were subtotally obliterated |  |
|  | 46 | M | Medial | Headache, tinnitus | MMA, OA, PMA | Mesencephalic BVR (varix) to VOG, cerebellar leptomeningeal vein | Borden III |  |  |  |
|  | 57 | F | Medial | Headache, tinnitus | MMA, OA, PMA | Mesencephalic BVR (varix) to VOG, cerebellar leptomeningeal vein | Borden II |  |  |  |
|  | 46 | M | Medial | Headache, myasthenia of limbs | SPA, MMA, OA | Cerebral leptomeningeal and cerebellar veins | Borden II |  |  |  |
|  | 62 | M | Medial | Headache (SAH), tinnitus, myasthenia of limbs | MMA, OA | Cerebral leptomeningeal venous aneurysm | Borden III |  |  |  |
|  | 42 | M | Medial | Headache | MMA, OA, SCA, PMA | Leptomeningeal veins | Borden III |  |  |  |
|  | 52 | M | Medial | Myasthenia of limbs | OA, SCA, MMA, SPA | Leptomeningeal veins | Borden III |  |  |  |
|  | 55 | M | Medial | Vertigo, seizure | MMA, OA, SPA, PMA | Leptomeningeal veins | Borden III |  |  |  |
|  | 57 | F | Medial | Headache | MMA, OA, PMA | Leptomeningeal venous aneurysm | Borden III |  |  |  |
|  | 43 | M | Medial | Headache, myasthenia of limbs | SPA, MMA, OA | Leptomeningeal and cerebellar leptomeningeal veins | Borden III |  |  |  |
|  | 66 | F | Medial | Headache, vomit, tinnitus, myasthenia of limbs | OA, SCA, MMA, SPA | Leptomeningeal veins | Borden III |  |  |  |
| Huang et al.^3^ | 59 | M | Galenic | IVH, hydrocephalus | OA, PCA | Vein of Galen (Varix) | III | None | Complete obliteration |  |
|  | 42 | M | Galenic | SAH/IVH | PCA, OA, MHT, MMA | Vein of Galen (Varix) | III | None | Near complete obliteration; recanalized at follow-up |  |
|  | 65 | M | Galenic | SAH | SCA (L), MHT (R), OA (L), APA | Perimesencephalic Vein of Galen | III | Microcatheter retention | Complete obliteration |  |
|  | 45 | M | Galenic | SAH | MMA, PCA, APA | Perimesencephalic versus cortical venous reflux | III | Vessel perforation | Failure |  |
|  | 59 | M | Galenic | SAH | PCA, MMA | Vein of Galen (Varix) | III | None | Partial obliteration; cure at follow-up |  |
|  | 40 | M | Galenic | SAH | MHT (L), MMA (L) | Vein of Galen (Varix) | III | None | Complete obliteration |  |
| Zhang et al.^4^ | 60 | M | Tentorium cerebelli | ICH | MMA (B), SCA (L) | Unknown | IV | SCA infarct | Complete obliteration |  |
|  | 41 | M | Tentorium cerebelli | Unknown | PCA (L) | Unknown | IV | SCA infarct | Complete obliteration |  |
| Wu et al.^5^ | 55 | M | Galenic | Hydrocephalus | MHT, PCA, MMA |  | IV | None | Complete obliteration |  |
|  | 43 | F | Straight sinus | SAH | PCA, MMA, MHT, PMA | Petrosal vein with cortical venous reflux | III | Intraoperative hemorrhage (from VA angiogram) | Complete obliteration | Pure pial (PCA) |
|  | 54 | F | Galenic | Headache | PCA, MMA, OA, ACA | VoG and BVR (varix) | IV | Intraoperative hemorrhage (from ACA) | Unknown | Pure pial (ACA) |
|  | 29 | M | Galenic | Headache | MHT, PCA, MMA |  | IV | None | Complete obliteration |  |
|  | 48 | M | Galenic | IVH | PCA, MMA, OA, PMA, MHT | VoG and BVR, cortical venous reflux and varix | IV | None | Complete obliteration |  |
| Byrne et al.^6^ | 45 | M | Falx cerebelli | Collapse, headache | MHT, ADS, SCA |  | IV | Died | Complete obliteration | Dilated dural branch (SCA) |
|  | 64 | M | Falx cerebelli | Incidentally | OA, MMA, PMA, MHT, SCA |  | IV | None | Complete obliteration | Dilated dural branch (SCA) |
|  | 64 | F | Torcula | Headache, progressive confusion, dysphasia | OA, MMA, ADS, SCA |  | IIa+b | None | Complete obliteration | Dilated dural branch (PCA,SCA) |
|  | 66 | M | Torcula | Visual disturbance and hemianopia | OA, PMA, MHT, ADS, SCA |  | IV | None | Subtotal obliteration | Dilated dural branch (PCA,SCA) |
|  | 60 | M | Falx cerebelli | Severe headache, vertigo | VA branches, PICA, SCA |  | III | None | Unchanged | Dilated dural branch (SCA) |
|  | 64 | F | Falx cerebelli | Collapse, vomiting, aphasia | OA, MMA, PMA, PICA, ADS |  | IV | Microcatheter rupture | Reduction | Dilated dural branch (PCA) |
| Fujii et al.^7^ | 43 | M | Tentorium cerebelli | SAH, IVH | ECA branches, bilateral MHT, SCA (R), AICA, PICA |  | IV |  | Reduction |  |
| Bhatia et al.^8^ | 30 | M | Falcotentorial | Headache, no SAH | ADS, MHT, recurrent meningeal |  | IV |  | Complete obliteration | Dilated dural branch (PCA) |
|  | 44 | F | Falcotentorial | Dizziness, headache, no SAH | ADS, MMA, OA, PMA |  | IV |  |  | Dilated dural branch (PCA) |
|  | 65 | M | Falcotentorial | Imbalance, no SAH | ADS, MMA, OA |  | IV |  |  | Dilated dural branch (PCA) |
|  | 53 | F | Falcotentorial | SAH | ADS, MMA, OA |  | IV |  |  | Dilated dural branch (PCA) |
| Gioppo et al.^9^ | 60 | F | Medial | Left progressive hearing loss and tinnitus | OA, MMA, PMA, tentorial branch from ICA (R), ADS |  | III | None | Complete obliteration | Dilated dural branch (PCA) |
| Choudhri et al.^10^ | 65 | F | Galenic | SAH from PICA aneurysm rupture s/p clipping | ADS, ABC, posterior choroidal branches |  | Iia+b | None | Complete obliteration | Dilated dural branch (PCA) |
|  | 62 | M | Tentorium cerebelli | Headache, photophobia, tinnitus | ADS etc.. |  |  | None | Complete obliteration | Dilated dural branch (PCA) |
| Zhou et al.^11^ | 35 | M | Tentorial incisura | Vertigo, diplopia, ataxia, CN V, VI, VII, VIII | MHT, MMA, SCA | Mesencephalic, BVR to VOG (varix), cerebellar venous aneurysm | III | None | Incomplete cure |  |
|  | 52 | F | Tentorial incisura | Tinnitus, SAH, CN V | MHT, MMA, PMA, PCA | Mesencephalic, BVR to VOG (varix), petrosal venous aneurysm | III | Cerebellar infarct | Complete obliteration after microsurgery |  |
|  | 29 | F | Lateral | Headache, CN VI, VII, III, ataxia | MHT, MMA, PCA, OA, PMA | Cerebral leptomeningeal and perimesencephalic (varix) spinal veins | III | None | Complete obliteration after microsurgery |  |
|  | 25 | M | Medial | Headache, diplopia, tinnitus, ataxia | MMA, PMA, PCA, SCA, OA | Cerebellar (varix) veins | III | None | Complete obliteration after microsurgery |  |
| Osada et al.^12^ | 48 | M | Tentorium (SPS) | Hydrocephalus |  |  | IV |  | Complete obliteration | Dilated dural branch (SCA) |
|  | 67 | M | Tentorium (SS) | Dizziness |  |  | IV |  | Complete obliteration | Dilated dural branch (SCA) |
|  | 53 | F | Tentorium (SS) | SAH |  |  | IV |  | Complete obliteration | Dilated dural branch (PCA, PICA |
|  | 70 | M | Tentorium (SS) | Gait and memory disturbance |  |  | III |  | Complete obliteration | Dilated dural branch (SCA) |
|  | 64 | M | Tentorium (SS) | Incidentally |  |  | IV |  | Unknown | Dilated dural branch (SCA) |
|  | 64 | F | Tentorium (Galenic) | Incidentally |  |  | IV |  | Reduction | Dilated dural branch (PCA, ACA) |
|  | 67 | M | Tentorium (Galenic) | Headache |  |  | IV |  | Unknown | Dilated dural branch (PCA, SCA) |
|  | 31 | M | Tentorium (Galenic) | Tinnitus, headache |  |  | IV |  | Obliterated | Dilated dural branch (PCA, SCA) |
|  | 58 | F | TSS | Tinnitus |  |  | IV |  | Obliterated | Pure pial (PCA) |
|  | 23 | M | TSS | Incidentally, post sinus thrombosis |  |  | IIa+b |  | Obliterated | Pure pial (PCA) |
|  | 41 | F | TSS | Tinnitus, headache |  |  | IIa |  | Reduction | Pure pial (PCA) |
|  | 67 | F | TSS | Tinnitus |  |  | III |  | Reduction | Pure pial (PCA); dilated dural branch (PICA) |
|  | 31 | F | TSS | Tinnitus, headache |  |  | IIa |  | Obliterated | Pure pial (PCA) |
|  | 44 | F | TSS | Tinnitus |  |  | IIa+b |  | Obliterated | Pure pial (PCA), dilated dural branch (SCA) |
|  | 57 | F | TSS | ICH |  |  | IV |  | Obliterated | Dilated dural branch (SCA, AICA) |
| Sato et al.^13^ | 40 | M | Tentorium cerebelli | Incidentally | MMA, OA, PCA (R) | Cortical veins | IV |  | SDH + ICH | Pure pial (PCA) |
| Jiang et al.^14^ | 42 | M | Tentorium | ICH, vertigo | MHT (R), MMA (R), SCA (R) | Cerebellar veins, PV, varix | IV | Trigeminocardiac reflex, hemifacial hypoesthesia, hemifacial palsy | Incomplete |  |
|  | 53 | F | Tentorium | ICH, vertigo | MMA (B), PCA (B), OA (B) |  | IV | None | Incomplete |  |
|  | 40 | M | Tentorium | ICH | MMA (L), MHT (L), SCA (L) | BVR, varix | IV | None | Obliterated |  |
|  | 42 | M | Tentorium | ICH | MMA (R), SCA (R), MHT (R) | PV, varix | V | None | Obliterated |  |
| Puffer et al.^15^ | 72 | M | Tentorium | Speech arrest | MMA (L), SCA (L), PMA (L) | Cortical vein, BVR, varix | III | None | Obliterated |  |
| Abbreviations: ABC, artery of Bernasconi-Cassanari. ADS, artery of Davidoff and Schechter. APA, ascending pharyngeal artery. BVR, basal vein of Rosenthal. CN, cranial nerve. ECA, external carotid artery. ICH, intracerebral hemorrhage. IVH, intraventricular hemorrhage. MHT, meningohypophyseal trunk. MMA, middle meningeal artery. OA, occipital artery. PCA, posterior cerebral artery. PICA, posterior inferior cerebellar artery. PMA, posterior meningeal artery. PV, petrosal vein. SAH, subarachnoid hemorrhage. SCA, superior cerebellar artery. SDH, subdural hemorrhage. SPA, sphenopalatine artery. SPS, superior petrosal sinus. SS, sigmoid sinus. STA, superficial temporal artery. TSS, transverse-sigmoid sinus. VOG, Vein of Galen. | | | | | | | | | | |

**Reference**

1. Hg K, Boukrab I, Bloemsma G, et al. Tentorial Dural Arteriovenous Fistulas : A Single-Center Cohort of 12 Patients. Published online 2017:284-290.

2. Liu C, Xu B, Song D, et al. Clinical approach of using Onyx via transarterial access in treating tentorial dural arteriovenous fistula. Published online 2014:983-991. doi:10.1179/1743132814Y.0000000383

3. Huang Q, Hong B, Liu J. CLINICAL STUDIES U SE OF O NYX IN THE M ANAGEMENT OF T ENTORIAL D URAL A RTERIOVENOUS F ISTULAE. 2009;65(2):287-293. doi:10.1227/01.NEU.0000348298.75128.D0

4. Zhang J, Lv X, Jiang C, Li Y, Wu Z. Superior cerebellar artery infarction in endovascular treatment for tentorial dural arteriovenous fistulas. 2010;74:33-37. doi:10.1016/j.ejrad.2009.03.048

5. Wu Q, Zhang XS, Wang HD, et al. Onyx Embolization for Tentorial Dural Arteriovenous Fistula with Pial Arterial Supply: Case Series and Analysis of Complications. *World Neurosurg*. 2016;92:58-64. doi:10.1016/j.wneu.2016.04.033

6. Byrne J V, Garcia M. Tentorial Dural Fistulas : Endovascular Management and Description of the Medial Dural-Tentorial Branch of the Superior Cerebellar Artery. Published online 2013.

7. Fujii N, Ideguchi M, Nishizaki T, et al. Case Report Successful treatment of a case of tentorial dural arteriovenous fistula causing subarachnoid hemorrhage with invagination of the brainstem by huge and multiple venous pouches. Published online 2019. doi:10.4103/sni.sni

8. Bhatia KD, Kortman H, Wälchli T, Radovanovic I, Pereira VM, Krings T. Artery of davidoff and schechter supply in dural arteriovenous fistulas. *Am J Neuroradiol*. 2020;41(2):300-304. doi:10.3174/ajnr.a6380

9. Gioppo A, Faragò G, Caldiera V, Caputi L, Cusin A, Ciceri E. Medial Tentorial Dural Arteriovenous Fistula Embolization: Single Experience with Embolic Liquid Polymer SQUID and Review of the Literature. *World Neurosurg*. 2017;107:1050.e1-1050.e7. doi:10.1016/j.wneu.2017.08.050

10. Choudhri O, Marks MP. Endovascular treatment of a tentorial dural arteriovenous fistula. *Neurosurg Focus*. Published online 2014. doi:10.3171/2014.V2.FOCUS14184

11. Zhou LF, Chen L, Song DL, Gu YX, Leng B. Tentorial dural arteriovenous fistulas. *Surg Neurol*. 2007;67(5):472-481. doi:10.1016/j.surneu.2006.08.078

12. Osada T, Krings T. Intracranial dural arteriovenous fistulas with pial arterial supply. *Clin Neurosurg*. 2019;84(1):104-114. doi:10.1093/neuros/nyy014

13. Sato K, Matsumoto Y, Endo H, Tominaga T. A hemorrhagic complication after Onyx embolization of a tentorial dural arteriovenous fistula : A caution about subdural extension with pial arterial supply. 2017;23(3):307-312. doi:10.1177/1591019917694839

14. Jiang C, Zhang J, Li Y. Transarterial Embolization of Intracranial Dural. Published online 2009. doi:10.3174/ajnr.A1419

15. Puffer RC, Daniels DJ, Kallmes DF, Cloft HJ, Lanzino G. Curative Onyx embolization of tentorial dural arteriovenous fistulas. *Neurosurg Focus*. 2012;32(5):1-5. doi:10.3171/2011.12.FOCUS11323
